# Soluble Molecularly Imprinted Nanorods for Homogeneous Molecular Recognition

**DOI:** 10.3389/fchem.2018.00081

**Published:** 2018-03-28

**Authors:** Rongning Liang, Tiantian Wang, Huan Zhang, Ruiqing Yao, Wei Qin

**Affiliations:** ^1^Key Laboratory of Coastal Environmental Processes and Ecological Remediation, Yantai Institute of Coastal Zone Research and Shandong Provincial Key Laboratory of Coastal Zone Environmental Processes, Chinese Academy of Sciences, Yantai, China; ^2^School of Chemical Engineering, Northwest University, Xi'an, China

**Keywords:** molecularly imprinted polymer, homogeneous recognition, nanorods, soluble polymer, polyanilines

## Abstract

Nowadays, it is still difficult for molecularly imprinted polymers (MIPs) to achieve homogeneous recognition since they cannot be easily dissolved in organic or aqueous phase. To address this issue, soluble molecularly imprinted nanorods have been synthesized by using soluble polyaniline doped with a functionalized organic protonic acid as the polymer matrix. By employing 1-naphthoic acid as a model, the proposed imprinted nanorods exhibit an excellent solubility and good homogeneous recognition ability. The imprinting factor for the soluble imprinted nanoroads is 6.8. The equilibrium dissociation constant and the apparent maximum number of the proposed imprinted nanorods are 248.5 μM and 22.1 μmol/g, respectively. We believe that such imprinted nanorods may provide an appealing substitute for natural receptors in homogeneous recognition related fields.

## Introduction

In nature, antibodies and enzymes play vital roles in various biological processes such as molecular recognition, signal transduction, and immune response (Baker, 2015; Schwalm et al., 2016). Despite their excellent specificities, these natural receptors are prone to denaturation in harsh environments, and difficult to prepare. During the past decades, the emergence of molecularly imprinted polymers (MIPs) has shown great promise to resolve these issues. MIPs are artificial receptors synthesized in the presence of the functional monomers, templates and cross-linkers by covalent, noncovalent or sacrificial spacer methods. Subsequent removal of the template yields the binding sites that are complementary in shape and size to the template (Haupt and Mosbach, 2000; Pan G. Q. et al., 2011). In recent years, tremendous progress has been made in this technology with MIPs generated for various targets ranging from small ions to larger proteins or even whole cells (Li et al., 2013, 2016; Shen et al., 2014). The ultimate goal of molecular imprinting is to generate artificial receptors with affinities and specificities approaching those of the biological receptors. Indeed, MIPs could eventually replace their biological counterparts in real applications (Pan J. M. et al., 2011; Cumbo et al., 2013). However, it should be noted that almost all of the recognition processes of natural receptors take place in homogeneous systems. Until now it is still a big challenge for MIPs to achieve homogeneous molecular recognition since they are highly cross-linked polymers and thus cannot be easily dissolved in organic or aqueous phase. This issue seriously limits the wide applications of MIPs in various areas such as homogeneous catalysis (Liu and Wang, 2010), chemical sensing (Liang et al., 2010, 2017), and medical treatment (Cutivet et al., 2009).

Up to now, very few soluble MIPs have been reported for homogeneous molecular recognition. The monomolecular imprinting inside dendrimers developed by Zimmerman's group is promising for the synthesis of soluble MIP (Zimmerman et al., 2002, 2003). However, this method requires elaborate syntheses and the selectivity of the obtained MIP is modest. Enzyme-like and soluble MIP nanogels synthesized by the dilution method was reported by Wulff et al. (2006). But the catalytic activity is rather low since the nanogels obtained by the dilution method are not dense enough to maintain a stable molecular memory. The much denser antibody-mimicking MIP nanogels, were synthesized by localized polymerization with multi-initiators (Çakir et al., 2013). However, the early termination reactions could occur by using a dendritic multi-initiator since several polymer chains are initiated in close proximity to each other. Very recently, our group described an approach for the synthesis of soluble MIP which was synthesized by the swelling of the traditional MIP at a high temperature (Zhang et al., 2018). However, such approach involves harsh synthesis conditions.

**Figure 1:**
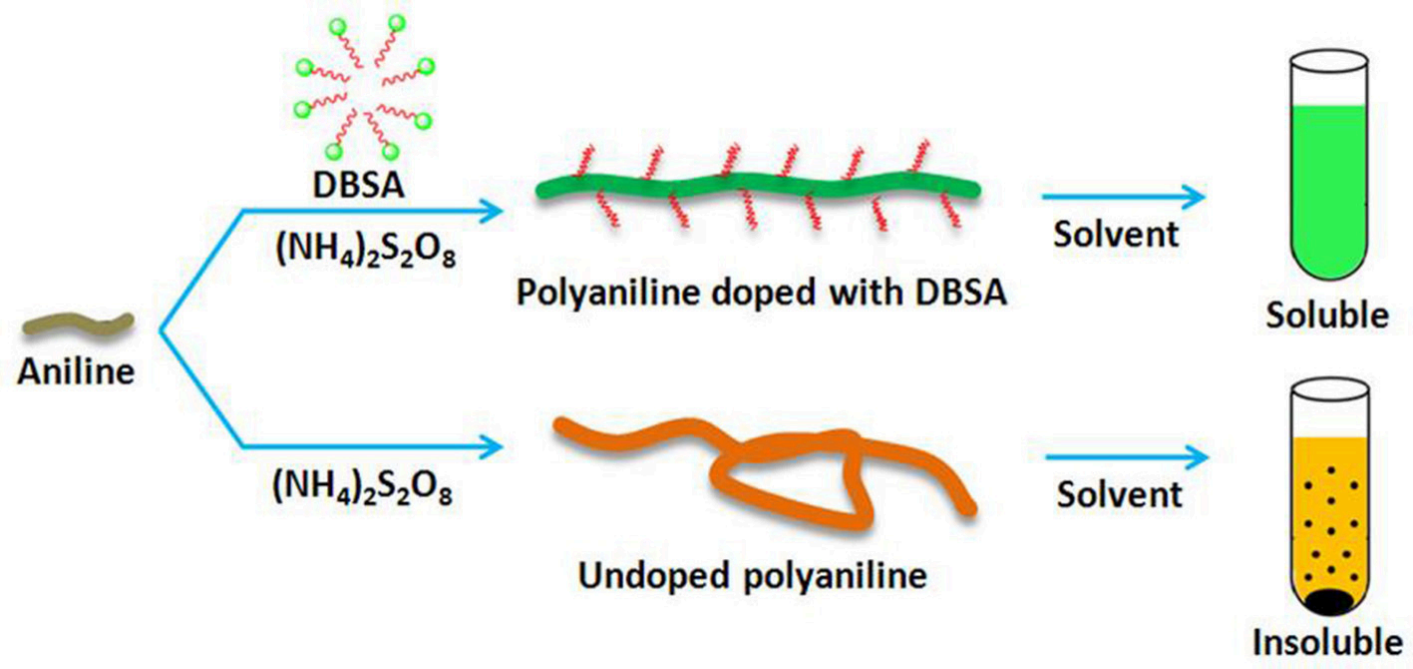
Representations of the syntheses of organic solvent-soluble and -insoluble polyanilines.

In this paper, we describe a novel method for the synthesis of soluble molecularly imprinted nanorods (MINs) with excellent binding affinity and specificity in organic phase. Soluble MINs can be synthesized by chemical oxidation of aniline monomer in the presence of the template. In order to obtain soluble polyaniline, a functionalized protonic acid such as dodecylbenzene sulfonic acid (DBSA) can be doped into the imine chains of polyaniline in polymerization (Cao et al., 1993). The long alkyl chains of the protonic acid can effectively enlarge the distance between the polyaniline molecule chains and further weaken the intermolecular interactions, thus significantly improving the solubilities of polyaniline in organic solvents (Figure 1; Lee et al., 2005, 2006). This paper reports a general and facile approach for the synthesis of soluble MIPs that exhibit efficient homogeneous recognition affinity in organic solvents. The proposed strategy may pay the way to developing MIPs with affinities approaching the natural receptors.

## Materials and methods

### Reagents

Aniline, acetonitrile, methanol, ethanol, chloroform, methylene chloride, and ammonium persulfate (APS) were obtained from Guoyao Chemical Reagent Co., Ltd (Shanghai, China). DBSA, 1-naphthoic acid (1-NA), 1-naphthalene methanol and 2-naphthalene methanol, naphthalene and phenol were purchased from Sigma-Aldrich. All the reagents were of analytical grade and used as received. Aqueous solutions were prepared by dissolving the appropriate salts in the freshly de-ionized water (18.2 MΩ cm specific resistance) obtained with a Pall Cascada laboratory water system.

### Synthesis of soluble MIP for 1-NA

Soluble MINs were synthesized by chemical oxidation of aniline monomers in the presence of the template. Briefly, the template 1-NA (2.0 g), the monomer aniline (4.7 g) and the dopant DBSA (19.6 g) were dissolved in ethanol (40 mL) and sonicated for 10 min to obtain homogeneity. Then 20 mL of the APS aqueous solution (2.5 mol/L) was added dropwise to the above solution for oxidation. Polymerization was carried out at room temperature for 6 h. After polymerization, the precipitate was filtrated and then washed with water until it was colorless. The template was removed by successive washing steps in a soxhlet extractor with acetonitrile until the template could not be detected at 218.5 nm by the high performance liquid chromatography (HPLC). The resulting polymer was dried in vacuum overnight at 50°C. Non-imprinted nanorods (NINs) were synthesized by the similar procedure in the absence of template molecules. The conventional insoluble MIP and non-imprinted polymer (NIP) were prepared under identical conditions except for omission of the dopant DBSA (see the Supplementary Material for the detailed procedures for the synthesis of conventional MIP and NIP).

## Results and discussion

**Figure 2:**
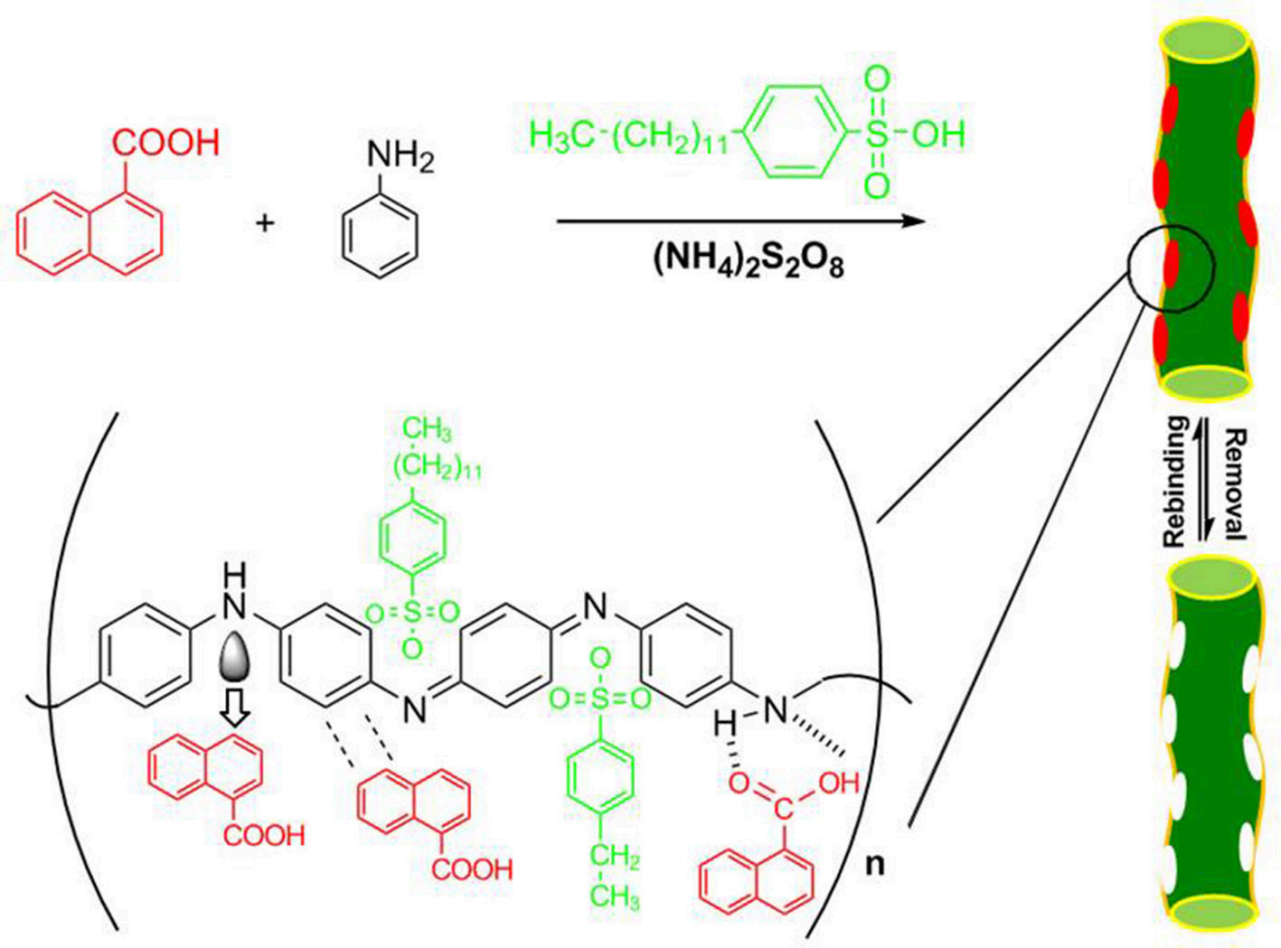
Schematic illustration of the synthesis of soluble MIN for 1-NA.

To demonstrate the general principle, we first chose noncovalent molecular imprinting system as a model and used 1-NA as the template which is a main degradation product of polycylic aromatic hydrocarbon naphthalene. The schematic representation of the principle is outlined in Figure 2. The soluble MIN was synthesized by chemical oxidation polymerization in aqueous solution in the presence of template 1-NA, monomer aniline, functionalized protonic acid DBSA, and the initiator ammonium persulfate. The soluble NINs were prepared with the same recipe except for omission of the template. As a comparison, the conventional insoluble MIP or NIP was prepared by the similar procedure in the absence of DBSA. The resultant polyaniline has plenty of noncovalent functionalities such as the imine group and the benzene ring which can provide the strong hydrogen binding and π-π interactions with the carboxyl group and the aromatic moiety of the template, respectively. Also, the charge-transfer complexing interaction may take place between the electron-deficient aromatic ring of 1-NA and the electron-rich imine group of polyaniline in solution system (Traven and West, 1973; Gao et al., 2007). These groups exactly match the characteristics of MIPs for efficient molecular recognition of 1-NA.

### Scanning electron microscope (SEM) characterization

**Figure 3:**
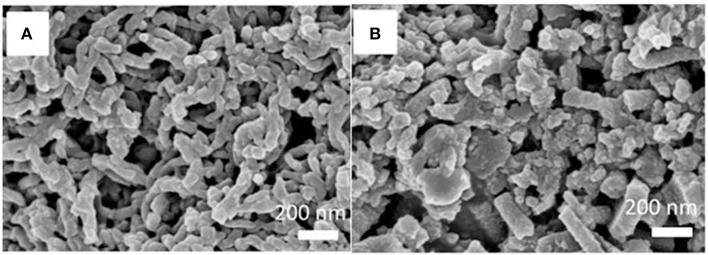
SEM images of the obtained soluble MIN **(A)** and insoluble MIP **(B)**.

The conventional insoluble MIPs and the soluble MINs were characterized by using SEM and the results are shown in Figure 3. Interestingly, significant differences in morphology and particle shape can be observed. As illustrated, the conventional MIPs without any stabilizer have a randomly distributed structure (Figure 3B), while the soluble MIN exhibits a uniform appearance with diameters of 70–90 nm (Figure 3A). This is probably due to the incorporation of DBSA which not only serves as a dopant for improvement of polyaniline solubility but also works as a surfactant for stabilization of polyaniline dispersion in polymerization (Kinlen et al., 1998; Haba et al., 2000). Indeed, the presence of DBSA can effectively prevent the undesirable side reactions and direct the growth of polyaniline one-dimensional nanostructures, thus leading to the formation of uniform nanorods. The lengths of the nanorods range from 200 nm up to several hundred nanometers. The nanorods tend to agglomerate into interconnected nanorod networks, rather than bundles. A closer look at the MIN shows that many of them are twisted. In addition, the SEM images also indicate that the soluble NINs prepared with the same recipe under both conditions have the similar morphological structure and particle size distribution with the MIPs (Figure S1).

### Fourier-transform infrared spectroscopy (FT-IR) spectrum

**Figure 4:**
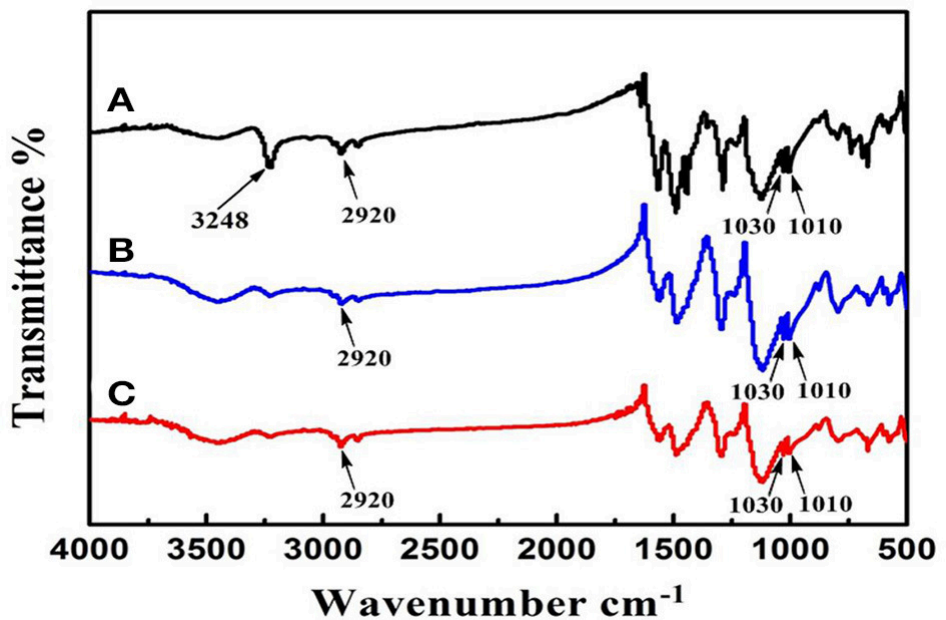
Infrared spectra of soluble MIN before **(A)** and after **(B)** extraction of 1-NA. Line **(C)** shows the spectrum of the control polymer (NINs).

In order to confirm the doping of DBSA and extraction of the template 1-NA, the infrared spectra were recorded. As shown in Figure 4, both the soluble NIN and MIN display the characteristic peaks of the aliphatic C-H stretching vibration of long alkyl tail of DBSA at the range of 2800–3000 cm^−1^, indicating that DBSA has been introduced into the polymer. Additionally, the characteristic peak of –COOH of 1-NA at 3248 cm^−1^ disappears after the extraction of the template molecules, which verifies the successful removal of the template. There is difference between curve a and curve b at 1480 cm^−1^ in Figure 4. In curve a, such peak can be ascribed to the skeleton vibration of the aromatic ring of the template 1-NA. After template removal, this peak disappears in curve b. This can further confirm that the template has been removed from the polymerized polymers after polymerization. After template removal, confirmation of the presence of DBSA in soluble MINs can be found in the Supplementary Materials.

### Solubility test

**Figure 5:**
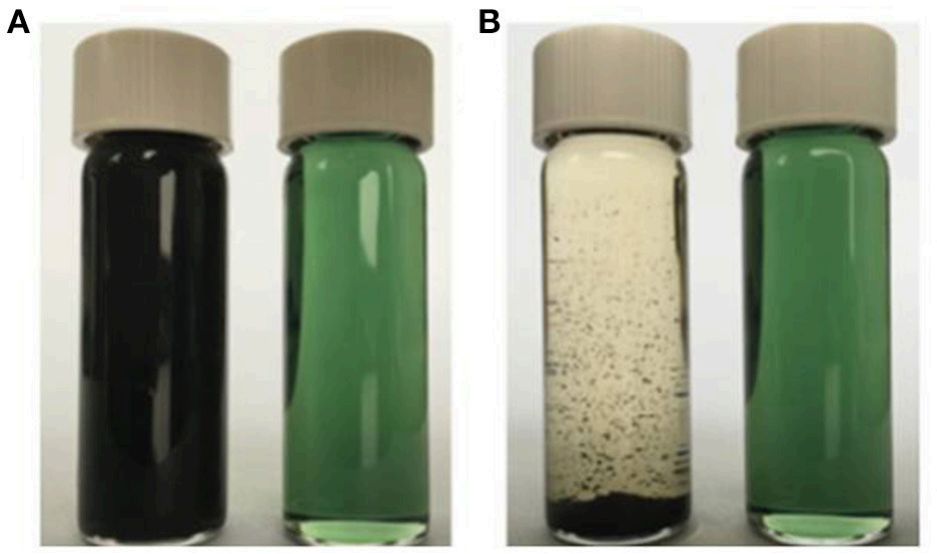
Dispersion stabilities of the conventional insoluble MIP and soluble MINs in CH_2_Cl_2_. The dispersions of the obtained polymers (5 mg/mL) in CH_2_Cl_2_ at ambient temperature are shown after ultrasonication for 5 min **(A)** and after settling down for 12 h **(B)**: left, insoluble MIP; right, soluble MINs.

To illustrate the solubilities of the resultant MINs in organic solvents, the solubility characteristics of the conventional MIP and the MINs were compared. A common solvent, CH_2_Cl_2_, was chosen as a model. Both polymers were firstly ultrasonicated in CH_2_Cl_2_ at room temperature for 5 min and then allowed to stand for 12 h. The results are shown in Figure 5. As expected, the proposed MINs can be dissolved in CH_2_Cl_2_ even at a high concentration level (5 mg/mL), and the MIN solution is still homogeneous after 12 h. Although the insoluble MIP can be well dispersed in the solvent after ultrasonic treatment, obvious precipitate is observed after standing for 12 h. These results suggest that the MINs have an excellent solubility in CH_2_Cl_2_. Note that, in our preliminary studies, the proposed MINs were also well dissolved in other organic solvents such as CHCl_3_, DMF, and DMSO. Such excellent solubilities in organic solvents can be reasonably attributed to the doping of DBSA with a long alkyl chain in the MIP structures. In addition, since the synthesis procedures for NINs and NIPs are same as those for MINs and MIPs, it can be expected that the solubility characteristics of these NIPs are also similar to those of their imprinted counterparts.

Note that, the experimental factors such as the temperature and the polarity of the solvent can also affect the solubility of the proposed MINs. In this early work, these experimental factors were not taken into consideration. Hence, there is significant scope to further develop such work to achieve better solubility performance. These efforts are currently in progress in our laboratory.

### Measurement of recognition ability

**Figure 6:**
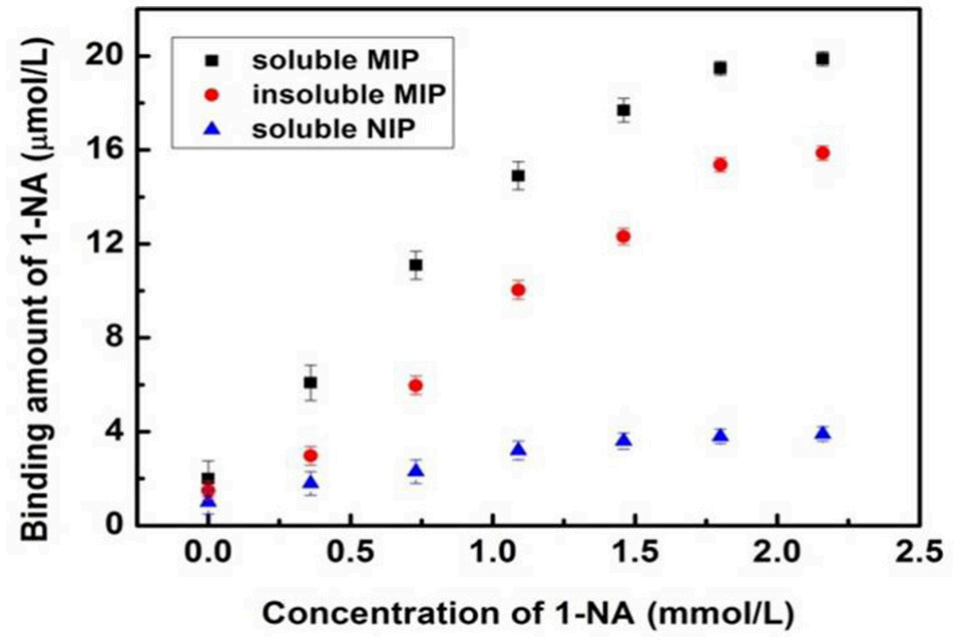
Equilibrium binding isotherms for the uptakes of 1-NA by the soluble MINs (■), traditional insoluble MIP (•) and soluble NINs (▴) in CH_2_Cl_2_. Error bars represent one standard deviation for three measurements.

The recognition ability of the soluble MINs for 1-NA was investigated by using the classical steady-state binding method (Ye et al., 2000; Umpleby et al., 2001). The binding isotherm of the insoluble MIP was tested as described before (Wei et al., 2015). Since the proposed MINs were fully soluble in solution, it cannot be easily separated from the incubation solution. In order to achieve the measurement of the binding isotherm, the MINs were isolated from the completely transparent solution by precipitation with acetonitrile after incubation. After filtration, the amount of the template 1-NA adsorbed by the soluble MIN was determined by measuring the residual 1-NA in filtrate by HPLC. The recognition ability of the control nanorods (NINs) were measured in the same manner. As shown in Figure 6 and Figure S2 in the Supplementary Material, both of the imprinted polymers (i.e., the conventional MIP and the soluble MIN) exhibit much higher capacities than the control polymers (soluble NIN and insoluble NIP) for 1-NA at all concentration ranges. These results suggest that the functionalities of 1-NA are responsible for the imprinting effect of MIP. More importantly, the binding ability of the soluble MIN is similar to that of the conventional insoluble MIP, suggesting that the imprinting efficiency is not affected by the doping of DBSA. Moreover, the MIN exhibits a slightly higher capacity than the conventional MIP. This is likely due to the small diameter of the nanorods that gives rise to a high surface area within the polymer network that can be accessed by the target. The imprinting factor for the MIN was calculated to be 6.8 according to the maximum binding amounts of the MIN and NIN obtained from the two response curves, thereby confirming the significant binding affinity of the MIN toward the target 1-NA. Furthermore, the Scatchard equation (Lenain et al., 2012) was employed to evaluate the relation between the concentration and binding ability, and the equation is expressed as:

$$\begin{array}{l} \frac{\text{Q}}{\text{C}}=-\frac{1}{{\text{K}}_{\text{D}}}\text{Q}+\frac{{\text{Q}}_{\text{max}}}{{\text{K}}_{\text{D}}} \end{array}$$

where Q stands for the binding capacity (μmol/L) of 1-NA on MIN, K_D_ represents the equilibrium dissociation constant (μmol/L), Q_max_ (μmol/g) is the theoretical maximum adsorption amount of template molecules on the MIN, and C (mol/L) is the equilibrium concentration of 1-NA in the solution. In the case of one ligand and one type of binding site, a straight line would be generated with a fixed slope rather than a curved one according to the Scatchard equation. Therefore, the values of Q_max_ and K_D_ should be considered as good indicators for the evaluation of the binding ability of the MIP. The application of the Scatchard equation to the calculations of the values for the binding isotherms renders the plot and the corresponding values for the apparent Q_max_ and K_D_ (Q_max_ = 22.1 μmol/g, K_D_ = 248.5 μM). The MIN curve shows a linear slope, indicative of one type of binding site possessing high affinity (Figure S3).

### Selectivity test

**Figure 7:**
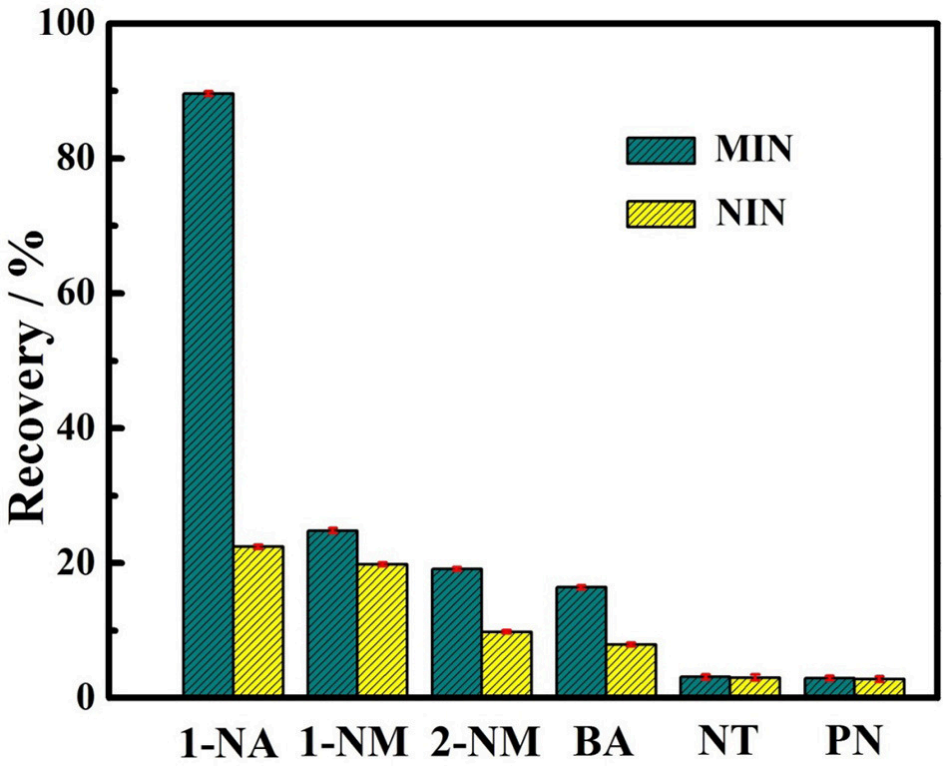
Binding recoveries of soluble MIN and NIN toward the template 1-NA and the interferents including 1-naphthalene methanol (1-NM), 2-naphthalene methanol (2-NM), benzoic acid (BA), naphthalene (NT), and phenol (PN). Error bars represent one standard deviation for three measurements. The concentrations of 1-NA and other interferents are 0.1 mM.

The selectivity test of the 1-NA-imprinted nanorods was carried out under equilibrium binding conditions using 1-naphthalene methanol (1-NM), 2-naphthalene methanol (2-NM), benzoic acid (BA), naphthalene (NT), and phenol (PN) as the analogs. The binding assays were carried out by incubating the soluble MIN or NIN in CH_2_Cl_2_ containing 1-NA or the analogs at the same concentration, respectively. As shown in Figure 7, the soluble MIN exhibits the highest binding recovery for 1-NA. Notably, the recoveries for the naphthalene derivatives such as 1-NM and 2-NM which have the same general structure as naphthalene but differ only in their functionalities are much lower than that for 1-NA. In addition, relatively lower binding recoveries of both MIP and NIP for benzoic acid which also has a carboxyl group can be observed compared to those for the template 1-NA. These results suggest that the MINs have a specific template binding ability. In sharp contrast, the control polymer shows the lower binding recoveries not only toward the template but also toward the interferents. The lower recoveries of the NIN are probably caused by the nonspecific adsorption of the polymer matrix. This further confirms the imprinting effect of the MIN.

## Conclusions

In summary, we have reported for the first time a general and facile approach for the synthesis of a fully soluble MIP in organic solvent with an excellent homogeneous binding capability and selectivity toward the template. The proposed MINs are based on soluble polyaniline doped with an organic protonic acid DBSA as the polymer matrix. Since polyaniline is a conducting polymer (Chiang and MacDiarmid, 1986), it can be expected that such polyaniline-based MIN will possess the advantage of superior conductivity while the conventional MIPs which are usually highly cross-linked polymers have low conductivity. This characteristic is very favorable for its application in electronic and optical applications. In addition, it should be noted that water soluble polyaniline can be easily synthesized by doping of –COOH or –NH_2_ group in the chains of polyaniline (Chen and Hwang, 1994). Thus, the proposed approach should be applicable to the synthesis of water soluble MINs based on polyaniline through doping of suitable hydrophilic groups. Given the universality and simplicity of this approach, it can be demonstrated that such MIN has the promising potential to ultimately replace corresponding biological receptors in many analytical applications such as catalysis, environmental monitoring, and clinical diagnosis.

## Author contributions

RL and WQ: designed the project; TW and HZ: conducted the experiments; RL, RY, and WQ: discussed and analyzed the results and jointly wrote the paper.

### Conflict of interest statement

The authors declare that the research was conducted in the absence of any commercial or financial relationships that could be construed as a potential conflict of interest.
