# Supplementary material for: Soluble Molecularly Imprinted Nanorods for Homogeneous Molecular Recognition
Source: Front Chem. 2018 Mar 28;6:81. doi: 10.3389/fchem.2018.00081 (PMC5890108; doi:10.3389/fchem.2018.00081)
Supplement: Supplementary file 1 [file DataSheet1.DOCX]

Supplementary Material

Soluble Molecularly Imprinted Nanorods for Homogeneous Molecular Recognition

RongningLiang^‡,a^, Tiantian Wang^‡b^, Huan Zhang^‡b^, Ruiqing Yao*^,b^ and Wei Qin*^,a^

*^a^ Key Laboratory of Coastal Environmental Processes and Ecological Remediation, Yantai Institute of Coastal Zone Research (YIC), Chinese Academy of Sciences(CAS); Shandong Provincial Key Laboratory of Coastal Environmental Processes, YICCAS, Yantai, Shandong 264003, P. R. China.*

*^b^ School of Chemical engineering, Northwest University, Xi^’^an 710069, P. R. China.*

^‡^ Rongning Liang, Tiantian Wang and Huan Zhang contributed equally to this work.

*Corresponding author. Tel.: +86-535-2109156, Fax: +86-535-2109000. E-mail address: yaoruiqing@nwu.edu.cn ; wqin@yic.ac.cn

***1.1 Batch binding experiments***

50 mg of the dry soluble nanorods (MINs or NINs) was placed in a 10 mL glass flask, and mixed with 2 mL of 1-NA in CH_2_Cl_2_ at different concentrations. The flask was sealed and shaken under agitation for 24 h. After incubation, 5 mL of acetonitrile was added to precipitate the nanorods. The nanorodrs were removed by centrifugation at 10,000 rpm for 5 min and the supernatants were collected. The amounts of 1-NA in the supernatant were determined by UV/Vis absorption spectroscopy at 218.5 nm. The amount of adsorbed 1-NA, expressed as percentage, was calculated by subtracting the final concentration from the initial concentrations of 1-NA in solution. The batch binding experiment for the conventional insoluble MIP was accomplished by the similar procedure except for omission of the procedure for nanorod precipitation.

The recovery was calculated by subtracting the amount of unbound one from the initial amount added to the mixture and then dividing by the initial amount:

$$\%R=\frac{C_{\text{initial amount}}-C_{\text{free amount}}}{C_{\text{initial amount}}}$$

***1.2 Selectivity test***

The selectivity test was done by incubating 50 mg of the soluble nanorods (MINs or NINs) in 2 mL of CH_2_Cl_2_ containing 1-NA or its analogues, at the same concentration (1.0×10^-4^ M) in a 10 mL polypropylene microcentrifuge tube. Then the tube was shaken under agitation for 24 h. After that, 5 mL of acetonitrile was added to precipitate the nanorods. The supernatant was collected. The amounts of free 1-NA were calculated by subtracting the final concentration from the initial concentration of 1-NA in CH_2_Cl_2_. The recovery was calculated by subtracting the amount of unbound one from the initial amount added to the mixture and then dividing by the initial amount.

***1.3 High-performance liquid chromatography (HPLC) measurements***

The HPLC measurements for 1-NA were performed by using a reversed-phase HPLC system which consists of a AIIiance e2695 (Waters, U.S.) pump, a photo-diode array (PDA) detector (Waters 2695-2998, U.S.), and a Waters C-18 column (SunFire, 5 µm 4.6 × 250 mm). Acetonitrile/H_2_O (80/20, v/v) was used as mobile phase with a flow rate of 1.0 mL / min and the column temperature was 35 °C.

***1.4 Synthesis of conventional insoluble MIP and NIP***

For the synthesis of conventional MIP, the template 1-NA (2.0 g) and aniline (4.7 g) were mixed with ethanol (40 mL) and sonicated for 10 min to obtain homogeneity. Then, 20 mL of the APS aqueous solution (2.5 mol/L) was added dropwise to the above solution for oxidation. Polymerization was performed at room temperature for 6 h. After polymerization, the precipitate was filtrated and then washed with water until it was colorless. Removal of the template was done by washing the polymer successively in a soxhlet extractor with acetonitrile. The resulting polymer was dired in vacuum overnight at 50 °C. Non-imprinted polymers (NIPs) were synthesized by the similar procedures except for omission of the template.

***1.5 Presence of DBSA in soluble MINs***

The proposed soluble MINs were synthesized in the presence of aniline, template and dopant DBSA by chemical oxidation. The synthesis procedures of soluble MINs are similar to those of DBSA-doped polyaniline. The mechanism for the synthesis of polyaniline doped by DBSA is shown as follows:


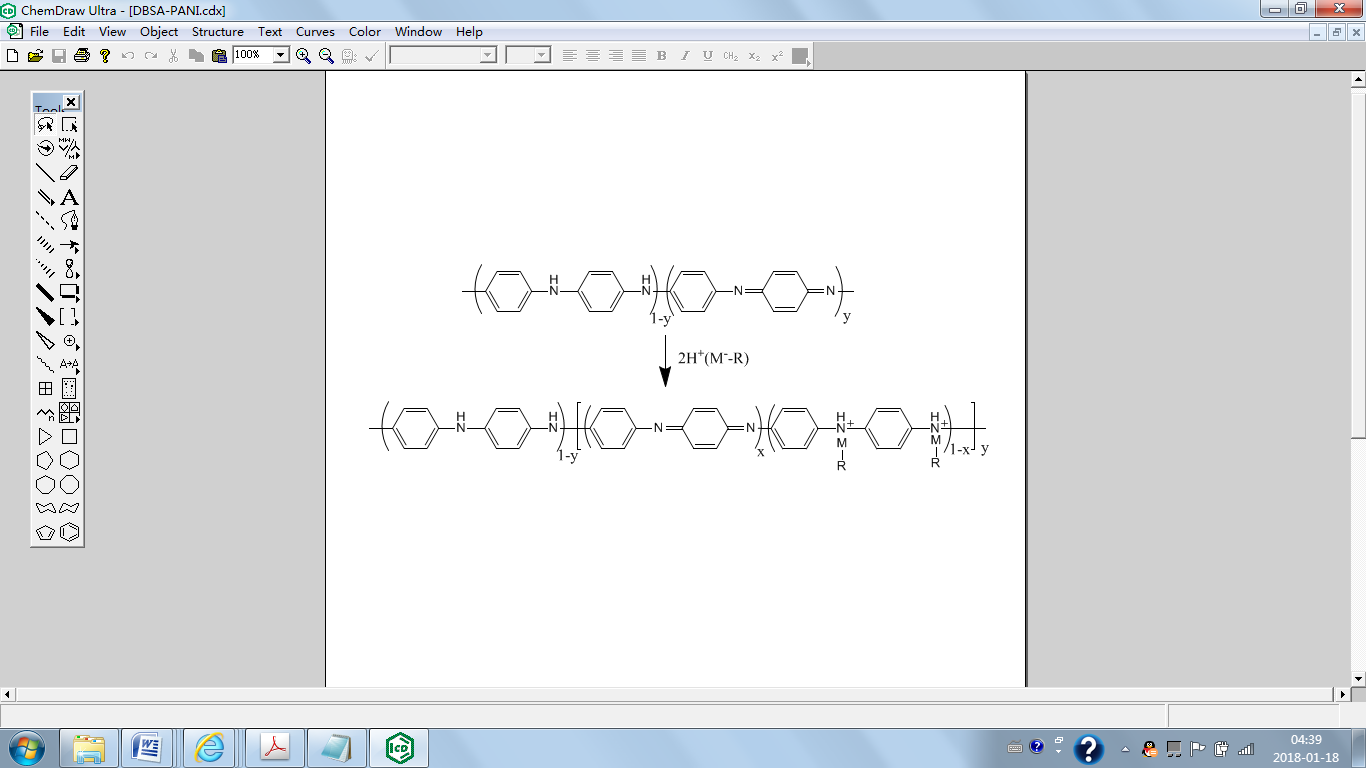


Here, H^+^(M^-^-R) stands for the organic protonic acid DBSA. As illustrated, DBSA is bonded to the backbone of polyaniline.^1,2^ In addition, after polymerization, the obtained DBSA-doped polyaniline is usually washed by some organic solvents such as acetone or methanol.^2,3^ Thus, it can be demonstrated that doped DBSA cannot be removed from the polymerized polymers by the template removal operations.

In addition, in the FT-IR spectrum of proposed soluble MINs (Figure 4), peaks at 2920, 1030, and 1010 cm^−1^ can be found in curves a, b and c, which correspond to S=O stretching, C-H stretching of CH_2_ and >CH stretching of benzenoin rings in the DBSA molecule. These peaks can also be found in the spectrum of DBSA.^2^  Therefore, it can be confirmed that DBSA is indeed in the polymer.

**References for the Supplementary Material**

1. Cao, Y., Smith, P. and Heeger, A. J. *Synth. Met.*, 1993, 55-57, 3514-3519.

2. Pan, W., Yang, S. L., Li, G. and Jiang, J .M. Eur Polym J., 2005, 41, 2127-2133.

3. Bhadra, J., Madi, N. K., Al-Thani, N. J. and Al-Maadeed, M. A. *Synth. Met.*, 2014, 191, 126-134.

**
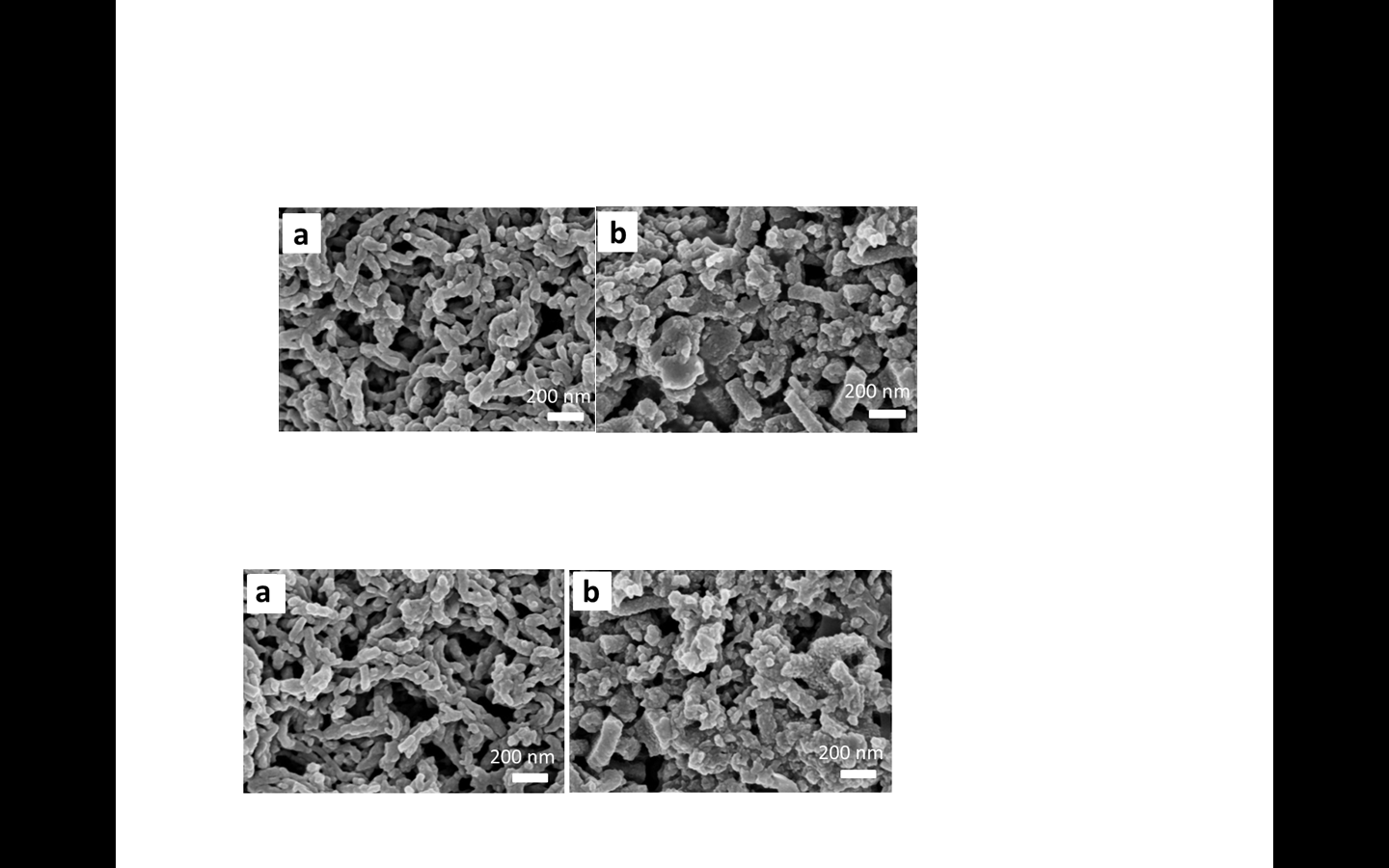
**

Supplementary Figure S1. SEM images of the obtained soluble NIN (a) and insoluble NIP (b)

**
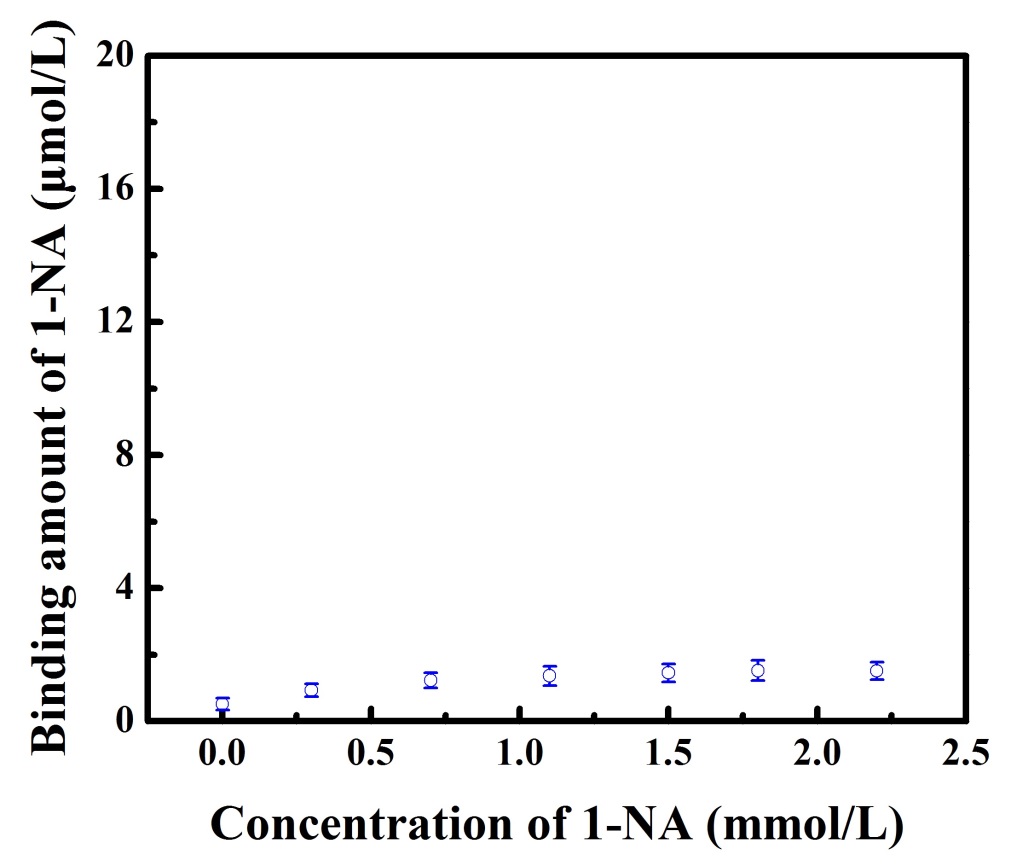
**

Supplementary Figure S2. Equilibrium binding isotherms for the uptakes of 1-NA by the traditional insoluble NIP in CH_2_Cl_2_. Error bars represent one standard deviation for three measurements.


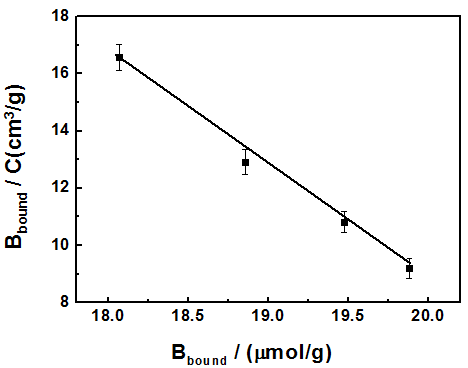


Supplementary Figure S3. Scatchard plot of the soluble MIN isotherm
